# Neuroprotective Effect of Oridonin on Traumatic Brain Injury via Inhibiting NLRP3 Inflammasome in Experimental Mice

**DOI:** 10.3389/fnins.2020.557170

**Published:** 2020-11-13

**Authors:** Chaolong Yan, Huiying Yan, Jiannan Mao, Yutong Liu, Li Xu, Hongting Zhao, Jiaqi Shen, Yan Cao, Yongyue Gao, Kuanyu Li, Wei Jin

**Affiliations:** ^1^Department of Neurosurgery, Nanjing Drum Tower Hospital, Clinical College of Nanjing Medical University, Nanjing, China; ^2^Department of Neurosurgery, Nanjing Drum Tower Hospital, The Affiliated Hospital of Nanjing University Medical School, Nanjing, China; ^3^Jiangsu Key Laboratory for Molecular Medicine, Medical School of Nanjing University, Nanjing, China

**Keywords:** traumatic brain injury, NLRP3 inflammasome, oridonin, neuroprotection, anti-inflammation

## Abstract

NLRP3 inflammasome has been considered as an important contributor to inflammation and neuronal death after traumatic brain injury (TBI). Oridonin (Ori), the major active ingredient of Chinese herbal medicine *Rabdosia rubescens*, has been proved to be a covalent NLRP3 inhibitor with strong anti-inflammation activity. The purpose of this study was to investigate the effect of Ori on inflammation and brain injury induced by TBI. Adult male C57BL/6 mice were subjected to closed-head injury using Hall’s weight-dropping method. Ori was injected directly intraperitoneally at a dose of 10 mg/kg within 30 min after TBI and injected once daily until the experiments ended. Our results showed that NLRP3 inflammasome was activated within 24 h post-TBI. The expression of NLRP3 inflammasome components (NLRP3, ASC, and caspase-1) was significantly decreased after treatment with Ori. Besides, the secretion of IL-1β and IL-18, downstream inflammatory factors of activated caspase-1, was reduced by Ori treatment. Importantly, Ori administration further protected the blood–brain barrier, alleviated brain edema, reduced cortical lesion volume, decreased cell death, and attenuated neurological deficits after TBI. Our findings indicate that NLRP3 inflammasome participated in the secondary injury after TBI and the application of Ori may provide neuroprotection via inhibiting NLRP3 inflammasome in animal models, suggesting that Ori might be a promising candidate for patients with TBI.

## Introduction

Traumatic brain injury (TBI) is a major cause of morbidity and mortality in the young population due to contact sports, motor vehicle accidents, or warfare (Garcia and Lopez-Rodriguez, 2019). Despite considerable progress in the diagnosis and therapy, the clinical prognosis of TBI patients remains unsatisfactory. The pathogenesis of TBI includes irreversibly primary mechanical injury and multifactorial secondary brain injury (SBI). A body of mechanisms are involved in SBI, including oxidative stress, neuroinflammation, calcium homeostasis, and apoptosis (Xu et al., 2018). Among these, neuro-inflammation has always been the vital focus of studies investigating the pathological development of TBI (Simon et al., 2017). Moderate inflammation is essential for cleaning cellular debris and facilitating neural repair, whereas excessive inflammation could aggravate neuronal damage and result in the exacerbation of pathological diseases (Lyman et al., 2014). Therefore, preventing the uncontrolled inflammation might alleviate SBI.

Convincing data have implicated a role of inflammation and subsequent cell death in the development of SBI (Jin et al., 2008; Corps et al., 2015). Inflammatory signaling is upregulated in both TBI patients and experimental TBI animals, e.g., the expression of nucleotide-binding oligomerization domain-like receptors (NLRs), nuclear factor-kappa B, interleukin (IL)-1β, and tumor necrosis factor-α were increased in cortex tissue and cerebrospinal fluid (CSF) (Hutchinson et al., 2007; Jin et al., 2008; Liu et al., 2013; Mettang et al., 2018). NLR family activation has been proved to trigger inflammatory responses and lead to poor outcomes after TBI (Mortezaee et al., 2018). The severity of early inflammation was related to poor Glasgow Coma Scale score in TBI patients on admission, accompanied by later blood–brain barrier (BBB) integrity disruption, brain edema, and long-term neurobehavioral deficits (Quagliarello et al., 1991; Holmin and Mathiesen, 2000; Tasci et al., 2003). Although the precise mechanisms about the inflammation after TBI are still not thoroughly clear, the result that inhibition of inflammation could attenuate SBI has been proved in TBI murine models (Ismael et al., 2018). What is more, activation of cell death signals after TBI such as pro-apoptotic pathway or pyroptotic death pathway also contributed to the progress of SBI (Sagulenko et al., 2013). Hence, reducing the inflammation and subsequent cell death may be of prime importance for patients suffering from TBI.

NLRs, one subclass of pattern recognition receptors (PRRs), play a critical role in the innate immune/inflammatory response by forming inflammasomes (Shao et al., 2015). Among the various inflammasomes, NLR pyrin domain containing 3 (NLRP3) inflammasome was the most widely studied and it has been regarded as a regulator of neuroinflammation in several central nervous system (CNS) disorders like Alzheimer’s disease (AD), Huntington’s disease, and pneumococcal meningitis (Jha et al., 2010; Hoegen et al., 2011; Tan et al., 2013; Zhou et al., 2016). NLRP3 inflammasome, a cytosolic signaling complex, consists of the sensor protein NLRP3, the adaptor protein apoptosis-associated speck-like protein containing a caspase recruitment domain (ASC), and the precursor enzyme pro-caspase-1. Upon stimulation by various stimuli (e.g., intracellular ionic fluxes, reactive oxygen species, and lysosomal damage) after tissue damage, components of NLRP3 inflammasome initiated assembly to form a multimeric molecular signaling platform, promoting the activation of caspase-1. Activated caspase-1 then proceeded to cleave the cytokine precursors, pro-IL-1β and pro-IL-18, into mature IL-1β and IL-18 (Yang et al., 2019). As pro-inflammatory cytokines, IL-1β and IL-18 initiated and amplified the inflammatory cascade after TBI (Woodcock and Morganti-Kossmann, 2013). Moreover, activated caspase-1 could provoke cell death directly by pyroptosis pathway or indirectly by pro-apoptotic pathway (Sagulenko et al., 2013). In a previous research in our laboratory, we have found the high expression of the NLRP3 inflammasome after TBI in animal models (Liu et al., 2013). Thus, blocking agents or inhibitors of NLRP3 inflammasome might be promising therapeutic targets to the inflammation after TBI.

Oridonin (Ori), a bioactive diterpenoid extracted from Chinese herbal medicine *Rabdosia rubescens*, is an NLRP3-selective inhibitor (He et al., 2018). In this study, it has been proved that Ori forms a covalent bond with cysteine 279 of NLRP3 in NACHT domain to block the interaction between NLRP3 and NEK7, thereby inhibiting NLRP3 inflammasome assembly and activation. Ori has also been reported to attenuate inflammation in various diseases such as rheumatoid arthritis, colitis, and AD (Wang et al., 2014; Liu et al., 2016; Shang et al., 2016). Based on these, the purpose of the current study was to investigate whether Ori could alleviate SBI after TBI via inhibiting the activation of NLRP3 inflammasome.

## Methods

### Animals and Experimental Groups

All procedures were reviewed and approved by the Institutional Animal Care and Use Committee at Nanjing Drum Tower Hospital and in accordance with the guidelines of the National Institutes of Health on the care and use of animals. Adult male C57BL/6 mice (8–10 weeks old, 20–25 g) were housed in animal facilities under temperature-controlled conditions with a 12-h light/dark cycle and fed with free access to water and food.

A total of 120 mice were randomly separated into three groups: sham (*n* = 40), TBI + vehicle (*n* = 40), and TBI + Ori (*n* = 40). Oridonin (Selleck Chemicals, Houston, TX) was injected directly intraperitoneally within 30 min after TBI at a dose of 10 mg/kg, the applicable dose to produce neuroprotection in AD (Wang et al., 2014). Our preliminary experiments have also confirmed that Ori attenuated the behavioral function at a dose of 10 mg/kg evaluated by modified neurological severity scoring (mNSS) in TBI-induced mice. However, Ori did not further attenuate the behavioral function at higher doses (20/40 mg/kg) compared with the dose of 10 mg/kg (Supplementary Figure S1).

After TBI induction, mice in TBI + Ori group were injected with Ori every day until the experiments ended. Six mice from each group were randomly selected for the analysis of Western blotting, RT-PCR, ELISA, brain water content, HE staining, Nissl staining, and TUNEL assays at day 1 after TBI and 15 mice for behavioral evaluation (mNSS, Rota-rod tests, and hanging wire tests) at days 1, 2, 4, 7, and 14 after TBI.

### Closed Head Injury Model

The murine model of TBI induced by weight drop method was performed as described in our previous study with some modification (Jin et al., 2009). Briefly, mice were anesthetized by intraperitoneal injection with sodium pentobarbital (50 mg/kg). Then the mice were placed in a stereotaxic apparatus and a 3.5-mm craniotomy was made over the right parietal cortex between bregma and lambda with the dura intact. A little pillar of 2.5 mm diameter was placed at the hit point and a 40-g weight was released, falling from a height of 10 cm along a stainless-steel string, striking the pillar. After the injury, the scalp incision was sutured and the mice were treated for 3 min with 100% oxygen administration and then placed in heated cages to recover from anesthesia. The sham mice were subjected to identical anesthetized and craniotomy only without contusion. All operations were executed with strict aseptic technique. Representative pictures of sham and TBI brains are shown in the section “Results.”

### Tissue Preparation

Animals were euthanized 24 h after TBI. The mice were deeply anesthetized with sodium pentobarbital (100 mg/kg) and perfused through the left cardiac ventricle with normal saline (4°C) until effluent from the right atrium was clear. The brains were harvested on ice. For the analysis of Western blotting, RT-PCR, and ELISA, the tissue samples from TBI group mice located around the contusion site within 5 mm and the same cortex from sham group mice were collected and immediately stored in liquid nitrogen for further measurements. For histological evaluation, the mice were perfused with normal saline (4°C) followed by 4% buffered paraformaldehyde (4°C) and then the brains were immersed in 4% buffered paraformaldehyde (4°C) overnight for further study. The tissue samples were sectioned at 4 μm thickness with a microtome. The slices which covered from the frontal side to the occipital side away from the edge of the contusion site within 5 mm were used for the histological evaluation. All the slices were stained by two pathologists blinded to the experimental condition.

### Evaluation of Neurobehavioral Deficits

Neurobehavioral deficits were evaluated with the mNSS, Rota-rod tests, and hanging wire tests by an investigator who was blinded to the experimental grouping according to the previous publication (Xu et al., 2018).

The mNSS tests consist of different tasks to evaluate the motor (muscle status, abnormal movement), sensory (visual, tactile, and proprioceptive), balance, and reflex functions of mice as shown in Supplementary Table S1. The grades ranged from 0 to 18 (0 = normal; 18 = the highest deficit) and a lower score reflects a better performance. Any mice with neurobehavioral deficit before the surgery was excluded and included mice were tested at five different time points after TBI (days 1, 2, 4, 7, and 14).

The Rota-rod tests were carried out to estimate coordination and balance ability of mice post-TBI. Mice were placed on an accelerating Rota-rod apparatus (RWD Life Science, Shenzhen, China), and the acceleration of the rotating rod was 10 rpm/min. When mice fall off the rotating rod, time was automatically recorded for the trial. Mice were trained three times the day before the surgery and then tested in the machine after surgery once a day with three times tests to obtain the average latency time to fall on days 1, 2, 4, 7, and 14 post-TBI.

The hanging wire tests were performed to assess the grip strength and endurance. Briefly, mice were placed on a wire frame to grip it and then flipped the wire frame over with 80 cm height off the ground. The latency time to fall was recorded and each mouse repeated three times to calculate the average time.

### Western Blotting

Western blotting was performed as previously described (Yan et al., 2015). First, we rinsed the tissues thoroughly with PBS to clear away the blood. The total proteins were extracted from the cortex tissue by complete homogenization in lysis buffer (Thermo Fisher Scientific, Waltham, MA), which contained protease inhibitors. The quantitation of total protein was performed by a BCA Protein Assay Reagent Kit (Beyotime, China). For each sample, equal amounts of proteins were loaded and separated on a 10–15% SDS-polyacrylamide gel and then transferred onto nitrocellulose (NC) membranes for 90 min at 250 mA. The transferred membranes were blocked in 5% skim milk–TBST for 1 h at room temperature and then incubated with primary antibodies at 4°C overnight. The primary antibodies used were as follows: anti-NLRP3 (1:500; Santa Cruz Biotechnology, Santa Cruz, CA), anti-ASC (1:200; Santa Cruz Biotechnology), anti-caspase-1 (1:1,000; Cell Signal Technology, Danvers, MA), anti-cleaved-caspase-1 (1:500; Santa Cruz Biotechnology), anti-occludin (1:1,000; Abcam, Cambridge, MA), anti-claudin-5 (1:1,000; Invitrogen, Carlsbad, CA), anti-ZO-1 (1:1,000; Abcam), anti-cleaved-caspase-3 (1:1,000; Cell Signaling Technology), and anti-β-actin (1:5,000; Sigma-Aldrich, St. Louis, MO). Then, the membranes were washed three times in Tris-buffered saline containing 0.1% Tween 20 and incubated with horseradish peroxidase (HRP)–conjugated secondary antibody (1:10,000; Beyotime) for 1 h at room temperature. After the secondary antibody incubation, the membranes were washed again four times. The membranes were visualized by enhanced chemiluminescence Western blotting detection system (Thermo Fisher Scientific). The densities of the bands were quantified by using ImageJ software (NIH, United States) and normalized to β-actin. The results were expressed as a relative density ratio, which was calculated as target protein expression/β-actin expression and presented as the fold change relative to the sham group.

### Real-Time Quantitative PCR

Total RNA was extracted using Trizol reagent (Invitrogen), and the purity and quantity of RNA were detected by spectrophotometric analysis. The cDNA was synthesized on 1 μg of total RNA using Reverse Transcription (RT) reagent kit (Thermo Fisher Scientific, United States). Real-time quantitative PCR was performed on the PowerUp SYBR Green Master Mix kit (Thermo Fisher Scientific, United States). According to the manufacturer’s instructions, the PCR master mixture was a 20-μl reaction system containing 10 μl SYBR Green, 0.4 μl upstream primer, 0.4 μl downstream primer, 2 μl cDNA, and 7.2 μl RNase-free water, which was carried out in a 96-well block. The optimal conditions were 40 cycles of 95°C for 30 s, 60°C for 32 s, and 72°C for 30 s. Relative quantification was given by the CT values, determined for triplicate reactions of tissue samples for each gene. Total RNA concentrations from each sample were normalized by quantity of β-actin messenger RNA (mRNA), and the expression levels of target genes were evaluated by using the 2^–Δ^ ^Δ^ ^*Cq*^ method, which were finally presented as the fold change relative to the sham group. The primers used are shown as in Supplementary Table S2.

### Enzyme-Linked Immunosorbent Assay

Total protein lysates of the cerebral cortex surrounding the traumatic site were obtained using the same method as described in the section “Western blotting.” The levels of inflammatory cytokines (activated IL-1β and IL-18, downstream products of activated NLRP3 inflammasome) were quantified using specific ELISA kits for mouse cytokines according to the manufacturers’ instructions (Invitrogen). The cytokine content of the cerebral cortex was expressed as picograms per milligram of protein.

### Measurement of Brain Edema

The wet/dry weight method, a critical measure of cortex water content, was used to measure brain edema as previously described by our group (Jin et al., 2011). After the mice were sacrificed 24 h post-TBI, the brain tissue was quickly removed and separated into the left cerebral hemisphere (contralateral) and right cerebral hemisphere (ipsilateral). Brain cortical samples were harvested, and immediately weighed to obtain the wet weight (WW). Then the samples were dehydrated in an oven for 24 h at 110°C and reweighed to obtain the dry weight (DW). Percentage of cortex water content was calculated as (WW − DW)/WW × 100%.

### Detection of Lesion Volume

To detect the cortical lesion volume 24 h after TBI, mouse brain specimens obtained after execution were cut into 4-μm-thick sections at intervals of 500 μm covering the entire injured cortex and stained with H&E. The slices were imaged using a light microscope (Leica). The areas of the ipsilateral and contralateral hemisphere were calculated using NIH ImageJ software and the area of cortical lesion size on each slice was calculated by the area of (contralateral hemisphere-ipsilateral hemisphere). The total lesion volume was the summation of the lesion size of each slice multiplied by the interval distance, and the results are shown as the lesion volume/the contralateral hemisphere volume × 100%.

### Nissl Staining

Nissl staining was performed as referred to our previous publication (Yan et al., 2017). Sections were stained with 1% toluidine blue at 50°C for 20 min and then rinsed with double-distilled water. After dehydrating, the sections were mounted with permount. After staining, Nissl body presents blue, which is one of the characteristic structures of neurons. In normal neurons, Nissl bodies are relatively abundant and cells show relatively big cell bodies and big round nuclei, whereas damaged cells show a reduction in quantity and show shrunken cell bodies, condensed nuclei, and many empty vesicles. The percentage of survived neurons was calculated as a ratio of normal neurons/(normal neurons + damaged neurons) × 100%. Four random fields in each slice were chosen and the mean of the percentage in the four views was regarded as the data of each slice. A total of three slices from each animal were used for quantification and the mean from three slices was regarded as the value of each animal. Six mice from each group were detected. All the slices were conducted by two pathologists blinded to the experimental condition.

### Terminal Deoxynucleotidyl Transferase (TdT) dUTP Nick-End Labeling (TUNEL) Staining

The 4% paraformaldehyde-fixed, paraffin-embedded tissue samples were sectioned at 4 μm thickness with a microtome. The sections were scored for apoptotic cells by the TUNEL method (Jin et al., 2014). TUNEL assays were conducted following the instruction of the kit (In Situ Cell Death Detection Kit; Boehringer Mannheim, Germany) and our laboratory methods (Jin et al., 2009). TUNEL-positive cells were counted and analyzed by two pathologists blinded to the experimental condition. The extent of brain damage was evaluated by the apoptotic index, which was the average percentage of TUNEL-positive cells in each section counted in four cortical microscopic fields. A total of three slices from each animal were used for quantification and the mean from three slices was regarded as the value of each animal. Six animals per group were analyzed.

### Statistical Analysis

Software SPSS 22.0 (IBM, Armonk, NY, United States) was used for statistical analysis of the data. All data were presented as mean ± SD. All data were subjected to one-way ANOVA. Differences between experimental groups were determined by Fisher’s LSD post-test. Statistical significance was inferred at *p* < 0.05. All precise *p* values are shown in Supplementary Table S3.

## Results

### Ori Administration Attenuates Neurological Deficits After TBI

**FIGURE 1:**
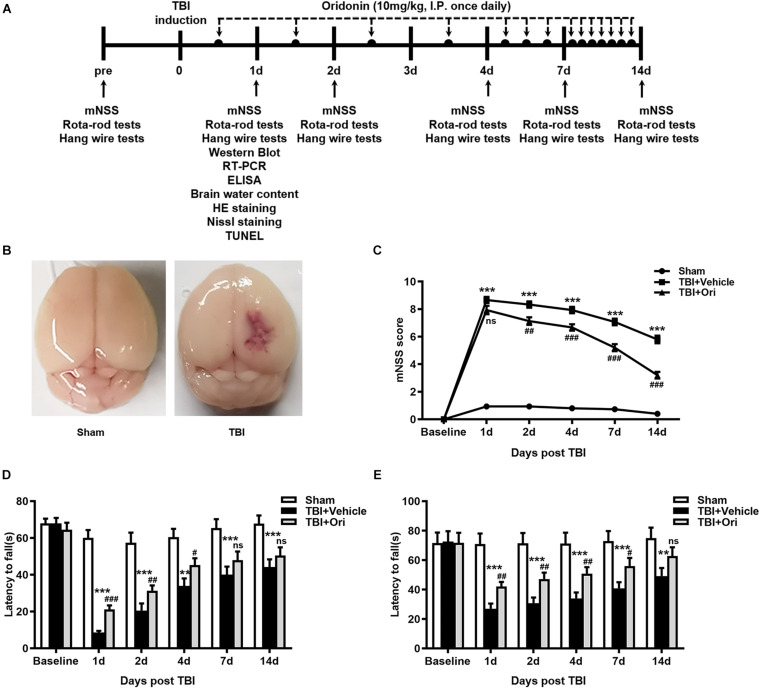
The effect of oridonin on neurological deficits after TBI. **(A)** Schematic diagram of the experimental design. **(B)** Representative photographs of sham and TBI brains. **(C–E)** Neurological performances were evaluated by the modified neurological severity scoring (mNSS) **(C)**, Rota-rod tests **(D)**, and hanging wire tests **(E)**. Oridonin treatment improved sensory-motor function, coordination function, and motor function compared with TBI + vehicle group. *n* = 15/group. Data are presented as the mean ± SEM. **p < 0.01 and ***p < 0.001 vs. sham group. ^#^*p* < 0.05, ^##^*p* < 0.01, and ^###^*p* < 0.001 vs. TBI + vehicle group. ^*ns*^*p* > 0.05.

To identify the protective effect of Ori on neurological deficits in mice, the mNSS, Rota-rod tests, and hanging wire tests were performed at different time points (days 1, 2, 3, 7, and 14) after TBI (Figure 1A). Representative pictures of sham and TBI brains are shown as in Figure 1B. Before surgery, all the animals were tested on these machines and the results showed that there was no difference among sham, TBI + vehicle, and TBI + Ori groups. The precise data of the overall neurological evaluation are shown in Supplementary Table S4. The mNSS tests results showed that TBI induced marked impairment of the motor and sensory function at all the time points (Figure 1C) and Ori-treated mice exhibited significant improvement at days 2, 4, 7, and 14 post-TBI compared with the vehicle-treatment mice (Figure 1C). Rota-rod tests showed the worst behavioral function in the TBI + vehicle group (Figure 1D) and significant improvement was observed after treatment with Ori. Similar performances were revealed with the hanging wire tests (Figure 1E). Thus, treatment with Ori improved the neurobehavioral performance and accelerated the recovery of mice after TBI, indicating the beneficial effects of Ori in TBI-induced injury.

### Ori Blocks the TBI-Induced Activation of NLRP3 Inflammasome

**FIGURE 2:**
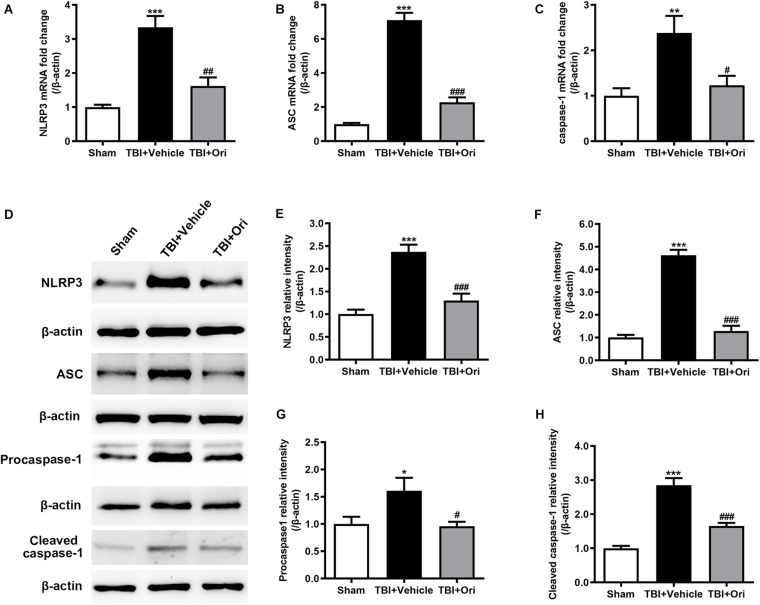
The effect of oridonin on the expression of NLRP3 inflammasome post-TBI. **(A–C)** Real-time quantitative PCR analysis for NLRP3, ASC, and caspase-1 in pericontusional cerebral cortex 24 h post-TBI. **(D–H)** Representative immunoblot bands and quantitative analysis of NLRP3, ASC, procaspase-1, and cleaved caspase-1 in pericontusional cerebral cortex 24 h post-injury. Oridonin treatment reduced the TBI-induced upregulation of NLRP3 inflammasome in the pericontusional cerebral cortex 24 h post-injury. *n* = 6/group. Data are presented as the mean ± SEM. *p < 0.05, **p < 0.01, and ***p < 0.001 vs. sham group. ^#^*p* < 0.05, ^##^*p* < 0.01, and ^###^*p* < 0.001 vs. TBI + vehicle group.

To determine the activation of NLRP3 inflammasome after TBI and evaluate the effect of Ori on inhibiting NLRP3, the expression of the components of NLRP3 inflammasome and cleaved activated caspase-1 was examined using RT-PCR and Western blotting. As shown in Figure 2, the expressions of NLRP3, ASC, and caspase-1 at both mRNA (Figures 2A–C) and protein levels (Figures 2D–H) were significantly increased after TBI, which was consistent with our previous study (Liu et al., 2013). Compared with the vehicle group, Ori treatment significantly decreased the upregulation of NLRP3, ASC, and caspase-1 at mRNA and protein levels 24 h after TBI. Administration of Ori further decreased the expression of cleaved caspase-1 in TBI mice, indicating that activation of caspase-1 was blocked. These results suggested that NLRP3 inflammasome was activated after TBI and Ori could significantly block the effect.

### Treatment With Ori Post-TBI Significantly Decreases the Expression of Inflammatory Cytokines in the Pericontusional Cerebral Cortex

**FIGURE 3:**
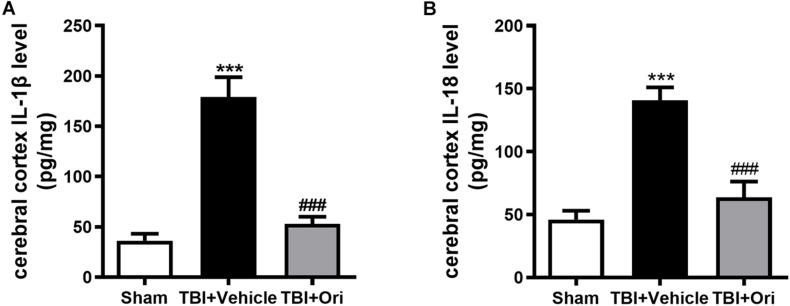
The effect of oridonin on the expression of subsequent inflammatory factors surrounding the injured cerebral cortex post-TBI. Concentration of inflammatory factors in pericontusional cerebral cortex was detected by ELISA 24 h post-TBI. The expression levels of activated IL-1β **(A)** and IL-18 **(B)** were significantly elevated in the TBI + vehicle group and the upregulation could be suppressed by the oridonin treatment. *n* = 6/group. Data are presented as the mean ± SEM. ***p < 0.001 vs. sham group. ^###^*p* < 0.001 vs. TBI + vehicle group.

Activated caspase-1 brings about the cleavage of pro-inflammatory factors IL-1β and IL-18, which were reported to increase at both mRNA and protein levels in different TBI models (Yang et al., 2019). To investigate whether the inhibition of NLRP3 by Ori could reduce the levels of pro-inflammatory factors, we tested the concentration of IL-1β and IL-18 in the cerebral cortex by using ELISA. In line with the data from previous studies, the concentrations of IL-1β and IL-18 in the pericontusional cerebral cortex were significantly elevated after TBI compared with the sham group (Figures 3A,B), whereas Ori treatment significantly inhibited the increase of IL-1β and IL-18 in the injured cortex area. Thus, the results demonstrated that Ori treatment suppressed NLRP3 inflammasome activation induced by TBI and reduced the levels of inflammatory cytokines, suggesting the association between the neuroprotective effect of Ori after TBI and the effect of Ori on anti-inflammation.

### Treatment With Ori Alleviates Cerebral Edema and Attenuates BBB Disruption After TBI

**FIGURE 4:**
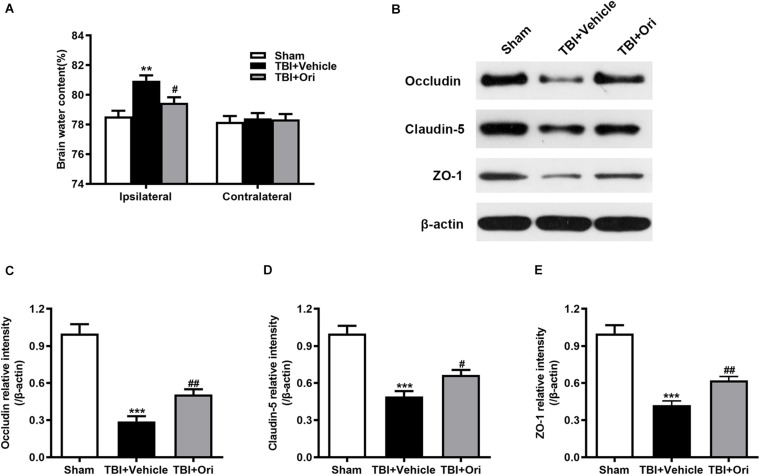
The effect of oridonin on the cerebral edema and BBB disruption after TBI. **(A)** The brain water content of ipsilateral and contralateral was detected 24 h post-TBI. Mice treated with oridonin showed remarkably decrease in brain water content in the ipsilateral hemisphere compared with TBI + vehicle group, whereas there was no significant difference in the contralateral hemisphere among the groups. **(B–E)** Representative immunoblot bands and quantitative analysis of occludin, claudin-5, and ZO-1 in the pericontusional cortex 24 h post-injury. Oridonin treatment improved the damage of BBB integrity after TBI. *n* = 6/group. Data are presented as the mean ± SEM. **p < 0.01 and ***p < 0.001 vs. sham group. ^#^*p* < 0.05 and ^##^*p* < 0.01 vs. TBI + vehicle group.

Wet/dry weight ratio method was used to examine the brain water content. As shown in Figure 4A, the brain water content in the damaged hemisphere was markedly increased in the TBI + vehicle group when compared with the sham group 24 h after injury. Treatment with Ori markedly alleviated cerebral edema (Figure 4A). This phenomenon was not observed in the uninjured hemisphere among the groups. The previous study has demonstrated that proinflammatory cytokines and chemokines released from the activated inflammatory cells are the critical factors contributing to BBB integrity disruption through decreasing the expression of tight junction proteins and increasing BBB permeability, eventually resulting in cerebral edema (Winkler et al., 2016). Therefore, we explored whether Ori has an effect on BBB protection by detecting the expression of tight junction proteins including occludin, claudin-5, and zonula occludens-1 (ZO-1). Western blotting revealed that TBI induced the decrease of levels of the tight junction proteins (occludin, claudin-5, and ZO-1) compared with the sham group and the treatment with Ori reversed the TBI-induced decrease (Figures 4B–E), indicating that treatment with Ori attenuated BBB disruption and alleviated cerebral edema after TBI. Thus, not only behavioral benefits but also the physiological protection are presented by Ori treatment.

### Treatment With Ori Reduces the Cortical Lesion Volume in the Traumatic Mice

**FIGURE 5:**
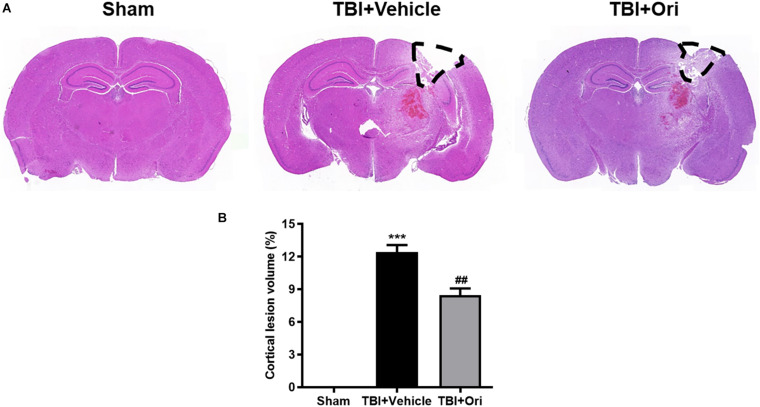
The effect of oridonin on the reduction of cortical lesion volume. H&E staining was used to evaluate the cortical lesion volume. **(A)** Representative images of slice from sham, TBI + vehicle, and TBI + Ori groups. No cortical lesion was observed in the cortex from sham mice whereas clear cortex loss or free was observed in TBI mice cortex (marked with black lines). **(B)** Quantification of the cortical lesion volume and shown as a ratio of the lesion volume to the contralateral hemisphere volume. Less cortical damage was detected in the mice treated with oridonin compared with vehicle after TBI. *n* = 6/group. Data are presented as the mean ± SEM. ***p < 0.001 vs. sham group. ^##^*p* < 0.01 vs. TBI + vehicle group.

Closed head injury model could cause a local cortical tissue lesion at the traumatic site. We evaluated the cortical lesion volume in the injured mice 24 h after TBI by H&E staining. As shown in Figure 5A, slices of sham group showed no cortical lesion while clear cortex loss was observed in the area of TBI mouse brain, where it was marked with black lines. By quantification of the cortical lesion volume, less cortical damage was detected in the mice treated with Ori compared with the vehicle mice after TBI (Figure 5B).

### Treatment With Ori Post-TBI Increases Neuronal Viability and Improves Neuronal Morphology

**FIGURE 6:**
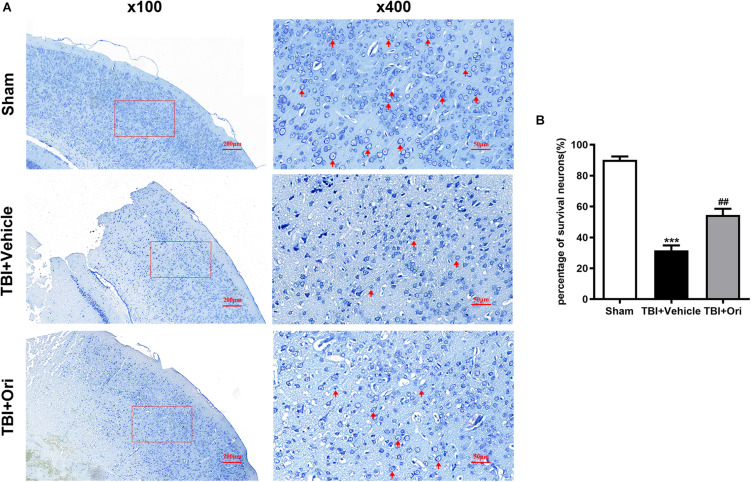
The effect of oridonin on the neuronal damage including cell quantity and structural changes after TBI. Nissl staining was used to evaluate the changes of cell quantity and morphology in mice cortical tissue. **(A)** Representative images of Nissl staining of slice from each group. Clear and intact neurons (marked with red arrow) were visible in the sham group, sparse arrangements and swollen bodies of cells were in the TBI + vehicle group, and oridonin treatment significantly improved the morphology of neurons. **(B)** Cell count was quantified in the slices. TBI reduced the number of surviving neurons, and the treatment with oridonin preserved neurons from loss after TBI. *n* = 6/group. Data are presented as the mean ± SEM. ***p < 0.001 vs. sham group. ^##^*p* < 0.01 vs. TBI + vehicle group.

The secondary injury after TBI is considered to lead to morphologic change and viability decline of neuron after inflammation. Nissl staining was performed to evaluate the quantity and morphology of the remained neurons in the cortex 24 h after TBI. Morphologically (Figure 6A), the visual field in the sham group was full of clear and intact neurons, whereas sparse cell arrangements, loss of integrity, and swollen cell bodies were observed in the traumatic injured mice. However, the presentation was significantly ameliorated in the Ori-treated group, confirming the neuroprotective effects of Ori after TBI. Besides, the average quantity of neurons was notably reduced around the traumatic injured area, which was partly reversed by Ori administration (Figure 6B).

### Treatment With Oridonin Attenuates the Neuronal Apoptosis in Mice That Suffered TBI

**FIGURE 7:**
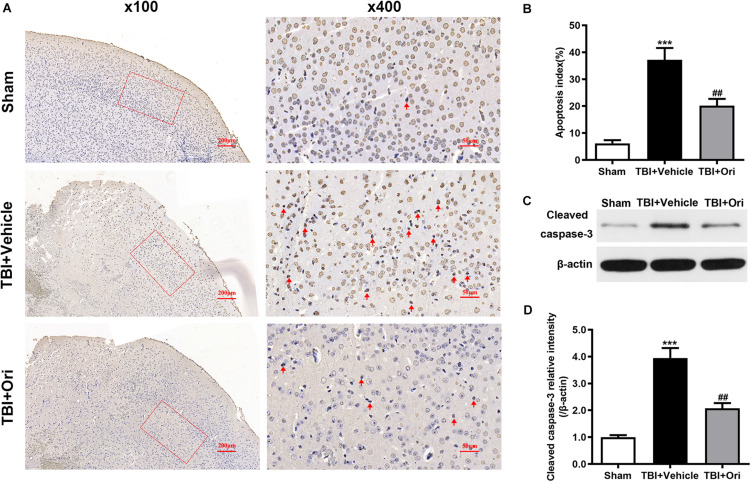
The effect of oridonin on the apoptosis after TBI in mice cortical tissue. **(A)** Representative images of TUNEL of each group. **(B)** Apoptosis index was calculated 24 h after TBI induction. Statistical data revealed that oridonin treatment significantly reduced the TUNEL-positive cells (marked with red arrow) compared with TBI + vehicle group. **(C,D)** Representative immunoblot bands and quantitative analysis of cleaved caspase-3 in the pericontusional cortex 24 h post-injury. Treatment with oridonin downregulated the increase of cleaved caspase-3 induced by TBI. *n* = 6/group. Data are presented as the mean ± SEM. ***p < 0.001 vs. sham group. ^##^*p* < 0.01 vs. TBI + vehicle group.

Cell apoptosis post-TBI is associated with NLRP3 inflammasome through an indirect pro-apoptotic pathway (Xu et al., 2018). To investigate whether the protective effects of Ori on neuronal survival are through this pathway, TUNEL assays (Figure 7A) were performed to detect the apoptotic cells and apoptotic index was used to evaluate the neuron death. Compared with the sham group, more apoptotic cells were detected in the TBI + vehicle group and Ori treatment significantly decreased the apoptotic index 24 h after TBI (Figure 7B). We also detected the expression level of cleaved caspase-3 using Western blotting (Figure 7C), which is a classic marker of apoptosis. As shown in Figure 7D, we found a significant increase of cleaved caspase-3 in TBI + vehicle group. Notably, administration of Ori reduced the level of cleaved caspase-3 compared with the TBI + vehicle group. Taken together, our results demonstrated that Ori could reduce the neuronal apoptosis after TBI via a caspase-3 mediated pathway and this protective role builds a rationale pharmaceutically in the treatment of TBI.

## Discussion

Ori has shown considerable anti-inflammatory activities in several CNS disorders (Wang et al., 2014). In the current study, our results have demonstrated that Ori may provide neuroprotective effects on TBI via inhibiting NLRP3 inflammasome. The main findings of our study are summarized as follows. First, administration of Ori inhibited the activation of NLRP3 inflammasome induced by TBI and reduced the levels of downstream proinflammatory cytokines. Second, Ori could markedly protect BBB integrity and reduce the brain edema after TBI. Third, treatment with Ori reduced cortical lesion volume, alleviated neuronal damage, and decreased the apoptosis following TBI and further attenuated the long-dated neurological deficits.

Accumulating studies have demonstrated that NLRP3 inflammasome plays a critical role in the pathogenesis of cell death after TBI. Among the triggers activating NLRP3 inflammasome after TBI, ionic fluxes, reactive oxygen species, and lysosomal damage are the three main signals (Jo et al., 2016). While stimulated by various endogenous or exogenous signals after brain injury, NLRP3 can recruit pro-caspase-1 and ASC to form a molecular platform for caspase-1 activation and subsequent maturation of IL-1β and IL-18. The release of pro-inflammatory cytokines IL-1β and IL-18 could further trigger the downstream inflammatory cascade, amplifying the inflammatory response by other pro-inflammatory pathways and aggravating cell death (Lu et al., 2005). Activation of NLRP3 inflammasome and subsequent release of IL-1β and IL-18 post-TBI have been reported in both pre-clinical and clinical studies. In our previous research, the expression of NLRP3, ASC, and cleaved caspase-1 increased in a time-dependent manner from 6 h to 7 days after injury at both mRNA and protein levels (Liu et al., 2013). IL-1β has also been reported to increase within a few minutes to hours post-TBI in CSF and brain parenchyma in both humans and animal models (Woodroofe et al., 1991; Frugier et al., 2010). Moreover, escalated NLRP3 was detected in CSF among children with severe TBI and was regarded independently related to poor prognosis (Wallisch et al., 2017). In the current study, we found that both protein and mRNA levels of NLRP3, ASC, and caspase-1 increased significantly 24 h after TBI and the concentration of pro-inflammatory cytokines were upregulated in pericontusional cerebral cortex 24 h post-injury, indicating the activation of the NLRP3 inflammasome and severe inflammation in mouse TBI models.

Due to the fact NLRP3 inflammasome is responsible for the severe neuroinflammation and cell death post-TBI, drugs targeting the inflammasome might be a potential therapeutic strategy for TBI by inhibiting NLRP3 inflammasome. Recently, several agents (Ori, propofol, omega-3 fatty acids, MCC950, etc.) have been proved to inhibit the activation of NLRP3 inflammasome through different ways (Ma et al., 2016; Lin et al., 2017; Xu et al., 2018). Ori, an active ingredient in traditional herb *Rabdosia rubescens*, was proved to suppress NLRP3-dependent inflammation as an NLRP3 inhibitor. Ori could perform strong anti-inflammation activity by covalent binding to cysteine 279 of NLRP3 in NACHT domain, blocking the interaction between NLRP3 and NEK7, which is the critical procedure to the activation of NLRP3 inflammasome. Through this combination, the assembly and activation of NLRP3 inflammasome could be inhibited consequently (He et al., 2018). In this study, we found intraperitoneal administration of Ori post-TBI could significantly inhibit the activation of NLRP3 inflammasome and reduce the secretion of downstream pro-inflammatory cytokines IL-1β and IL-18. Moreover, Ori administration dramatically attenuated neurological deficits within the 14-day observation period. Ori could exert a long-lasting neuroprotective effect in experimental TBI.

In addition to the inflammation, NLRP3 inflammasome also has been reported to cause both apoptotic and pyroptotic cell death via ASC (Sagulenko et al., 2013). What is more, IL-1β and IL-18 could further induce BBB integrity disruption, brain edema, and apoptosis (Holmin and Mathiesen, 2000). Cell death and intracranial hypertension induced by brain edema are associated with the neurological deficits and mortality after TBI (Stoica and Faden, 2010; Jha et al., 2019). In our study, brain edema after TBI was significantly alleviated around the injured brain tissue. More importantly, we further found that this protective effect might relate to alleviating BBB damage partly.

Apoptosis in the injured brain after TBI is a prominent form of cell death that occurs not only as programmed cell death of senescent cells but also as a result of SBI. In the present study, our data confirmed the occurrence of apoptosis post-TBI and the application of Ori could significantly decrease the percentage of apoptotic cells in the examined cerebral cortex. Accompanied by the anti-inflammation, administration of Ori alleviated cerebral edema, protected BBB integrity, reduced neuron apoptosis, and improved the neurobehavioral function.

Collectively, the current study demonstrated that Ori, a covalent inhibitor of NLRP3 inflammasome, effectively prevented the inflammatory response and neuronal apoptosis, protected BBB integrity, and attenuated neurological deficit in TBI mice. These data suggest that Ori might be a promising pharmaceutical candidate for future clinical trials. To strengthen the clinical relevance, further studies are needed to reveal the therapeutic window of Ori and more detailed mechanism of the neuroprotection.

## Conclusion

Activation of NLRP3 inflammasome initiated and amplified the inflammation induced by TBI and aggravated other SBI, resulting in eventual cell death. Treatment with the NLRP3 inflammasome inhibitor, Ori, attenuated inflammation, cell death, and neurological deficits after TBI. These results suggested that Ori might be a prospective therapeutic candidate for patients suffering from TBI.

## Data Availability Statement

The raw data supporting the conclusions of this article will be made available by the authors, without undue reservation.

## Ethics Statement

The animal study was reviewed and approved by the Institutional Animal Care and Use Committee at Nanjing Drum Tower Hospital.

## Author Contributions

CY and HY designed the project and wrote the article. JM and YG participated in the TBI model and analyzed the data of the animal studies. YL performed the Western blotting. HZ performed H&E staining and Nissl staining. LX performed TUNEL staining. JS performed RT-PCR. YC performed the ELISA. KL and WJ contributed to conception and provided critical revisions. All authors checked and approved the final article.

## Conflict of Interest

The authors declare that the research was conducted in the absence of any commercial or financial relationships that could be construed as a potential conflict of interest.
