# Supplementary material for: Neuroprotective Effect of Oridonin on Traumatic Brain Injury via Inhibiting NLRP3 Inflammasome in Experimental Mice
Source: Front Neurosci. 2020 Nov 13;14:557170. doi: 10.3389/fnins.2020.557170 (PMC7691250; doi:10.3389/fnins.2020.557170)
Supplement: Supplementary file 1 [file Data_Sheet_1.PDF]

Supplementary Materials

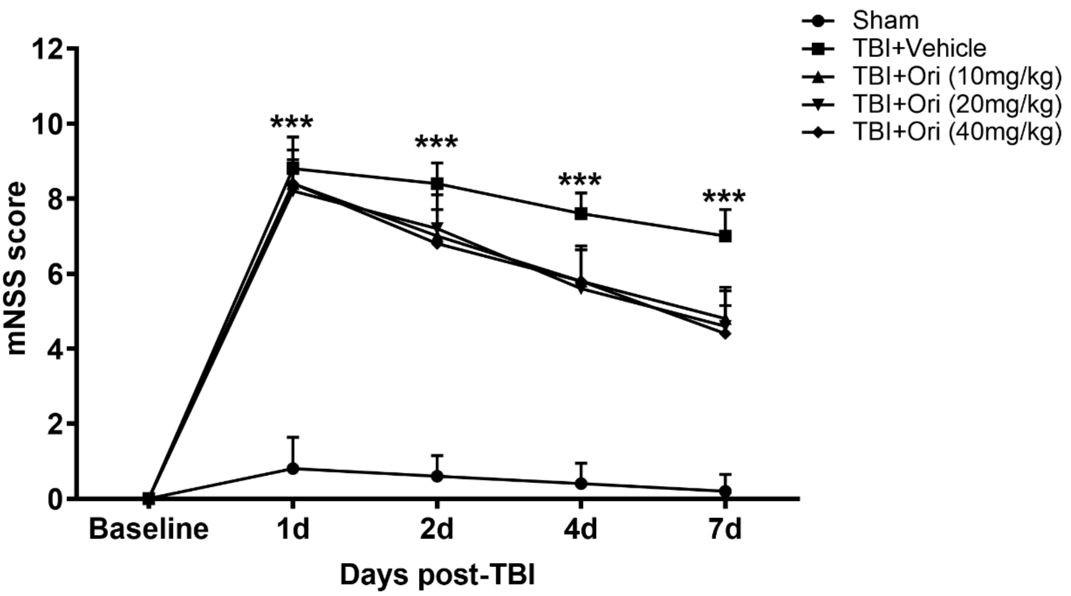

Figure S1. Different dosages of Ori all attenuated the behavioral function at the dosages of 10/20/40 mg/kg evaluated by mNSS in TBI-induced mice. However, Ori did not further attenuate the behavioral function at higher dose (20/40 mg/kg) compared with the dosage of 10 mg/kg. No clear difference was found between 10 mg/kg group and higher doses group (20/40 mg/kg).

Data are presented as the mean ± SEM. ***$p < 0.001$ vs. sham group.

Table S1. modified Neurological Severity Scoring (mNSS)

**Motor tests**	**6**
Raising rat by tail	3
Flexion of forelimb	1
Flexion of hindlimb	1
Head moved >10° to vertical axis within 30s	1
Placing rat on floor (normal=0; maximum=3)	3
Normal walk	0
Inability to walk straight	1
Circling toward paretic side	2
Falls down to paretic side	3
**Sensory tests**	**8**
Placing test (visual and tactile test)	1
Proprioceptive test (deep sensation, pushing paw against table edge to stimulate limb muscles)	1
Beam balance tests (normal=0; maximum=6)	6
Balances with steady posture	0
Grasps side of beam	1
Hugs beam and 1 limb falls down from beam	2
Hugs beam and 2 limb fall down from beam, or spins on beam (>60s)	3
Attempts to balance on beam but falls off (>40s)	4
Attempts to balance on beam but falls off (>20s)	5
Falls off; no attempt to balance or hang on to beam (<20s)	6
**Reflex absence and abnormal movements**	**4**
Pinna reflex (head shake when auditory meatus is touched)	1
Corneal reflex (eye blink when cornea is lightly touched with cotton)	1
Startle reflex (motor response to a brief noise from snapping a clipboard paper)	1
Seizures, myoclonus, myodystonia	1
**Maximum points**	**18**

Table S2. Primers of target genes used

Target genes	Primers
NLRP3	forward 5′-GAAGAAGAGTGGATGGGTTTG-3′<br>reverse 5′-CTGCGTGTAGCGACTGTTGAG-3′
ASC	forward 5′-TGCTTAGAGACATGGGCTTAC-3′<br>reverse 5′-CTGTCCTTCAGTCAGCACACT-3′
Caspase-1	forward 5′-GACAAGGCACGGGACCTATGT-3′<br>reverse 5′-CAGTCAGTCCTGGAAATGTGC-3′
β-actin	forward 5′-AGGGAAATCGTGCGTGAC-3′<br>reverse 5′-CGCTCATTGCCGA-TAGTG-3′

Table S3. The precise *p* values which were contained in the paper

p values / Experiments	Sham vs. TBI+Vehicle	TBI+Vehicle vs. TBI+Ori
mNSS		
Day 1	<0.0001	0.0636
Day 2	<0.0001	0.0024
Day 4	<0.0001	0.0006
Day 7	<0.0001	<0.0001
Day 14	<0.0001	<0.0001
Rota-rod tests		
Baseline	0.9933	0.4849
Day 1	<0.0001	<0.0001
Day 2	<0.0001	0.0330
Day 4	0.0002	0.0484
Day 7	0.0005	0.2295
Day 14	0.0005	0.3137
Hang wire tests		
Baseline	0.9316	0.9359
Day 1	<0.0001	0.0026
Day 2	<0.0001	0.0082
Day 4	0.0001	0.0091
Day 7	0.0004	0.0360
Day 14	0.0073	0.1062
RT-PCR		
NLRP3	<0.0001	0.0021
ASC	<0.0001	<0.0001
Caspase-1	0.0075	0.0239
Quantitative analysis of WB bands		
NLRP3	<0.0001	0.0007
ASC	<0.0001	<0.0001
Procaspase-1	0.0483	0.0264
Cleaved caspase-1	<0.0001	0.0004
Occludin	<0.0001	0.0048
Claudin-5	<0.0001	0.015
ZO-1	<0.0001	0.0019
Cleaved caspase-3	<0.0001	0.0011
ELISA		
IL-1β	<0.0001	0.0001
IL-18	<0.0001	0.0008
Brain water content		
Ipsilateral	0.0011	0.0173
Contralateral	0.6632	0.8882

Cortical lesion volume	<0.0001	0.0012
Percentage of survival neuron	<0.0001	0.0008
Apoptosis index	<0.0001	0.0076

Table S4 Precise data of the overall neurological evaluation

**Experiments**	**Sham**	**TBI+Vehicle**	**TBI+Ori**
mNSS (scores)			
Day 1	0.9333 ± 0.1533	8.667±0.2323	7.933 ± 0.3003
Day 2	0.9333 ± 0.1817	8.333 ± 0.2323	7.133 ± 0.2737
Day 4	0.8000 ± 0.1147	7.933 ± 0.2282	6.667 ± 0.2323
Day 7	0.7333 ± 0.1182	7.067 ± 0.2482	5.200 ± 0.2619
Day 14	0.4000 ± 0.1309	5.800 ± 0.2795	3.200 ± 0.2430
Rota-rod tests (s)			
Baseline	67.95 ± 2.561	67.92 ± 3.012	64.47 ± 3.826
Day 1	60.13 ± 4.151	8.647 ± 0.8369	21.21 ± 2.136
Day 2	57.45 ± 5.456	20.57 ± 3.879	31.34 ± 2.832
Day 4	60.47 ± 4.486	33.96 ± 4.065	45.29 ± 3.688
Day 7	65.47 ± 4.775	40.08 ± 4.396	47.97 ± 4.679
Day 14	67.87 ± 4.384	44.27 ± 4.093	50.49 ± 4.463
Hang wire tests (s)			
Baseline	71.67 ± 7.053	72.53 ± 7.093	71.73 ± 6.840
Day 1	70.93 ± 7.162	27.00 ± 3.539	42.20 ± 2.949
Day 2	71.40 ± 7.011	30.87 ± 3.764	47.20 ± 4.330
Day 4	71.27 ± 7.343	34.00 ± 4.051	50.80 ± 4.424
Day 7	72.93 ± 6.757	40.80 ± 4.183	56.00 ± 5.489
Day 14	75.00 ± 7.041	49.13 ± 5.515	62.73 ± 5.997
